# Disturbances in metabolic, transport and structural genes in experimental colonic inflammation in the rat: a longitudinal genomic analysis

**DOI:** 10.1186/1471-2164-9-490

**Published:** 2008-10-17

**Authors:** Olga Martínez-Augustin, Manel Merlos, Antonio Zarzuelo, María Dolores Suárez, Fermín Sánchez de Medina

**Affiliations:** 1 Department of Biochemistry and Molecular Biology II, CIBEREHD, School of Pharmacy, University of Granada, Campus de Cartuja s/n, 18071 Granada, Spain; 2 Palau Pharma, Pol. Ind. Riera de Caldes Avinguda Camí Reial 51-57, 08184 Palau-solità i Plegamans, Barcelona, Spain; 3 Department of Pharmacology, CIBEREHD, School of Pharmacy, University of Granada, Campus de Cartuja s/n, 18071 Granada, Spain

## Abstract

**Background:**

Trinitrobenzenesulphonic acid (TNBS) induced rat colitis is one of the most widely used models of inflammatory bowel disease (IBD), a condition whose aetiology and pathophysiology are incompletely understood. We have characterized this model at the genomic level using a longitudinal approach. Six control rats were compared with colitic animals at 2, 5, 7 and 14 days after TNBS administration (n = 3). The Affymetrix Rat Expression Array 230 2.0 system was used.

**Results:**

TNBS-induced colitis had a profound impact on the gene expression profile, which was maximal 5 and 7 days post-induction. Most genes were affected at more than one time point. They were related to a number of biological functions, not only inflammation/immunity but also transport, metabolism, signal transduction, tissue remodeling and angiogenesis. Gene changes generally correlated with the severity of colitis. The results were successfully validated in a subset of genes by real-time PCR.

**Conclusion:**

The TNBS model of rat colitis has been described in detail at the transcriptome level. The changes observed correlate with pathophysiological disturbances such as tissue remodelling and alterations in ion transport, which are characteristic of both this model and IBD.

## Background

Inflammatory bowel disease (IBD), comprising ulcerative colitis and Crohn's disease, is characterized by chronic and relapsing inflammation of the gastrointestinal tract. The pathogenesis of IBD is unknown, but it appears to be multifactorial in origin, and genetic, environmental and dietary factors are believed to be involved [1]. Animal models of IBD have been central to the investigation of the pathophysiology of the disease and are valuable tools for drug testing and development. Because IBD-like diseases do not occur spontaneously in animals, several animal models that mimic different aspects of the disease are currently used, including gene knockout, transgenic, chemical, adoptive transfer and spontaneous models [2]. To date, no single model has reproduced all features of the human disease. One of the most widely used models in both pharmacological and pathophysiological studies (72 in the past year) is murine colitis induced by trinitrobenzenesulphonic acid (TNBS) [3-5]. This simple model is based on a single rectal administration of TNBS dissolved in ethanol. TNBS is a hapten that elicits an immune response when bound to tissue proteins, while ethanol contributes to disruption of the intestinal barrier. The result is a severe and prolonged degenerative inflammation of large parts of the colon sharing several clinical and molecular characteristics with Crohn's disease. Specifically, the inflammation produced by the administration of TNBS-ethanol involves all layers of the intestinal mucosa and produces long-lasting damage with cell infiltration and ulcers, including protracted physiological dysfunction. Furthermore, both TNBS-ethanol administration to mice and human Crohn's disease are characterized by Th1-driven inflammation with infiltration of macrophages and neutrophils, producing high levels of proinflammatory cytokines such as tumour necrosis factor, interleukin (IL)-1β and IL-6, followed by T cell infiltration, mainly of the CD4+ phenotype.

Genomic profiling of disease models is of interest for characterizing the pathological response at transcriptome level and identifying putative drug targets. Animal models may overcome many of the limitations of the application of genomic technology to humans, including the need for repeated encoscopy, the large genetic and phenotypic variability, and the difficulty of studying the initial stages of the disease. There have been a few attempts at gene expression profiling in IBD models [6-10]. In general, these studies have addressed acute colitis (48–72 h after induction), employed small microarrays (containing 87 and 1252 transcripts in two of the studies), have analysed large samples (augmenting internal genomic variation, which occurs along the longitudinal axis) and include modest validation experiments (6–14 genes). Although a recent study by te Velde et al. [8] used 20,000 transcript microarrays, the data were not validated. Only one of the studies was longitudinal [9]. Therefore, the present study represents the most ambitious and comprehensive investigation to date, using several microarrays to examine the progression of colitis at four time points, employing a genechip platform that includes more than 30,000 transcripts (Affymetrix Rat 230 2.0). Results were validated in a subset of almost 100 transcripts by using real-time PCR (qRT-PCR). The full results database, publicly accessible, will serve as a valuable reference for all researchers in the field. In fact, three pharmacological studies adopting this strategy are currently underway in our laboratory.

## Methods

All reagents were obtained from Sigma (Barcelona, Spain) except where indicated.

### Animals

Female Wistar rats (175–225 g) were used, housed in makrolon cages and maintained in air-conditioned animal quarters with a 12-h light-dark cycle. Animals had free access to tap water and were fed a standard chow diet (Panlab A04, Panlab, Barcelona, Spain). This study was carried out in accordance with the Directive for the Protection of Vertebrate Animals used for Experimental and other Scientific Purposes of the European Union (86/609/EEC), was approved by the Ethical Committee of the University of Granada and complies with the American Physiological Society's Guiding Principles in the Care and Use of Animals.

### Induction of colitis

Colitis was induced as previously described [11]. Briefly, rats were fasted overnight and anaesthetized with halothane. Under these conditions, rats were given 10 mg of TNBS dissolved in 0.25 ml of 50% ethanol (v/v) by means of a Teflon cannula inserted 8 cm into the anus. Rats were kept in a head-down position for an additional 30 s and returned to their cages.

### Experimental design

Rats were randomly assigned to one of two different groups, a control (C, n = 6) group that received a saline enema and a TNBS group (n = 12) that received the TNBS challenge. Food and water intake and body weight were determined daily. To follow the progression of the colitis, three rats of the TNBS group were killed 2, 5, 7 and 14 days after the induction of colitis. Three control rats were killed on day 2 and the other three at the end (day 14) of the experiment. For the purpose of postgenomic validation, this experiment was repeated in order to perform qRT-PCR analysis on fresh samples. The magnitude and time course of the inflammatory response were similar in both experiments (data not shown).

### Assessment of colonic damage

Animals were killed by cervical dislocation, and the entire colon was removed and placed on an ice-cold plate, cleaned of fat and mesentery, and blotted on filter paper. Each specimen was weighed and its length measured under a constant load (2 g). The large intestine was longitudinally opened and scored for visible damage on a 0 to 25 scale as previously described [12]. A sample for genomic analysis was obtained from the distal colon approximately 4.5 cm proximal to the anus, taking care to avoid any areas of necrosis. The colon was subsequently divided longitudinally into several pieces for biochemical determinations. The fragments were immediately frozen in liquid nitrogen and kept at -80°C until used. Myeloperoxidase activity was measured according to the technique described by Krawisz et al. [13].

### RNA extraction, microarray hybridization and data analysis

RNA was extracted from homogenized full-thickness colonic tissues in Trizol^® ^reagent (Invitrogen) and purified with RNeasy affinity columns (Qiagen). Quantity and integrity of RNA were assessed by spectrophotometry and 0.8% agarose gel electrophoresis, respectively. Sample labelling, hybridization, staining and scanning procedures were carried out using Affymetrix standard protocols . The microarray analysis was performed by Progenika Biopharma (Bilbao, Spain) on 18 GeneChip^® ^Rat Expression Array 230 2.0 microchips (Affymetrix). Normalization and statistical analyses were carried out using GeneSpring v7.1 (Agilent). Gene ontology analysis was performed with GeneMapp/MappFinder [14]. Specifically, biological process, cellular component and molecular function categories were scored with respect to the number of genes included in the *Rattus norvegicus *database that were measured in the microarray and the number of genes significantly affected by TNBS colitis in each category. The resulting z parameter [15] has positive values when the proportion of genes affected is higher than expected, and it was used to select the most representative gene ontology categories (i.e. those with z ≥ 4) at each time point. Non-redundant categories are shown, listing only the highest z value when nested categories exceeded the cutoff value at multiple levels. The k-means algorithm was applied to identify and group transcript changes over time in clusters. Only sequences with annotated gene identities that were present (intensity > 100 units) in at least 66% of samples per group in at least one group were considered. The data were analyzed by analysis of variance followed by Tukey *post-hoc *tests in order to reduce the false positive or type I error rate in inter-group comparisons. In addition, the Benjamini & Hochberg false discovery rate correction was applied to reduce the occurrence of type I error when comparing among genes. This procedure offers a reasonable balance between sensitivity and specificity. Data are expressed as fold change (mean ± SEM) over the control (uninflamed) group (n = 6) at each time point (TNBS colitis after 2, 5, 7 or 14 days). MIAME recommendations [16] were followed to ensure that all information needed to understand, interpret, reproduce and compare our results was given in detail. The data are accessible at the the European Bioinformatics Institute Arrayexpress database (, reference E-MEXP-873).

### Postgenomic validation

Postgenomic validation was carried out by measuring 93 of the genes in fresh samples (n = 3 per group) using qRT-PCR with TaqMan^® ^Low Density Arrays (Applied Biosystems). Genes were selected to include both significantly and non-significantly changed genes pertaining to different families affected by inflammation, i.e., those related to transport, immunity or metabolism. The relative Ct values of each gene with respect to the reference gene (18S) were used to calculate the RQ (relative quantitation) parameter, which represents the change in mRNA expression compared to a control sample. The RQ was then used to calculate fold change ratios. Results are expressed as mean ± SEM.

## Results

### TNBS colitis

**Figure 1:**
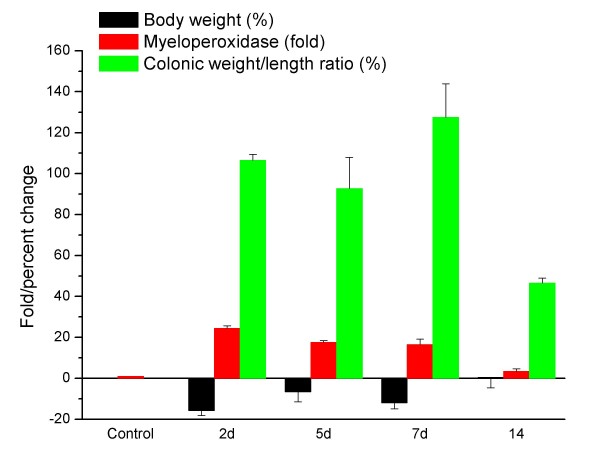
**Inflammatory biochemical and morphological markers in TNBS-induced colitis.** All means were different from the control values, except for body weight gain at 14 days (not shown).

The morphological and biochemical features of TNBS colitis were consistent with previous reports by our group and other authors [4,5,12,17]. Thus, TNBS-treated rats suffered anorexia and loss of body weight (Fig. 1) associated with extensive mucosal damage, oedema, haemorrhage and early epithelial necrosis. Leukocyte infiltration was prominent, resulting in a significant increase in myeloperoxidase activity (Fig. 1). Epithelial regeneration gradually occurred from day 7 and was macroscopically complete by day 14. At 5 and 7 days, there was major submucosal fibrosis and scarring that resulted in a marked shortening of colonic length. Even after 2 weeks, treated rats showed significant differences with controls in colonic weight-to-length ratio and myeloperoxidase activity, among others (Fig. 1).

### Genomic analysis

**Figure 2:**
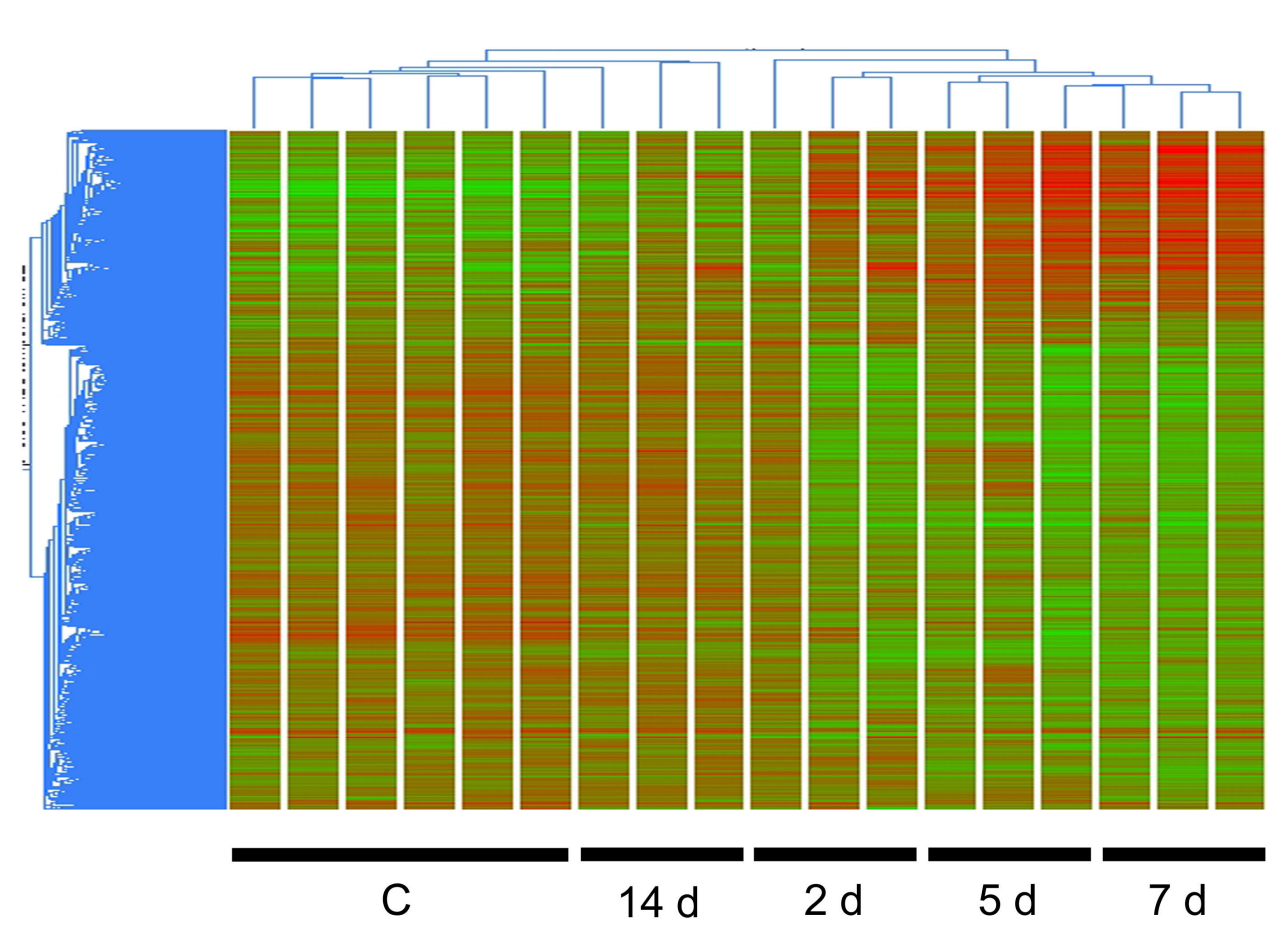
T**ime course changes of gene expression in rat TNBS colitis.** Sample clustering by two-way analysis of variance. Upregulated genes are shown in red and downregulated genes in green. The difference between TNBS colitis and control samples was greatest on day 7.

2340 genes were significantly modified at 2 days after TNBS instillation (1238 upregulated, 1102 downregulated). This number was almost doubled at day 5 (4266, 2073 of which were upregulated) and rose to 5752 (2356 upregulated) by day 7. The pool of altered gene expression fell to 1953 by day 14 (1146 upregulated), coinciding with a marked recovery from the inflammatory bout. The dendogram in Figure 2 shows that samples from TNBS colitic rats at each time point were clustered together, forming groups that significantly differed from control samples. As expected, many of the genes that showed significant changes are directly related to the inflammatory response, including genes for chemokines, cytokines and inflammatory markers such as Cp, Ptgs2, or Lyz (see below).

**Figure 3:**
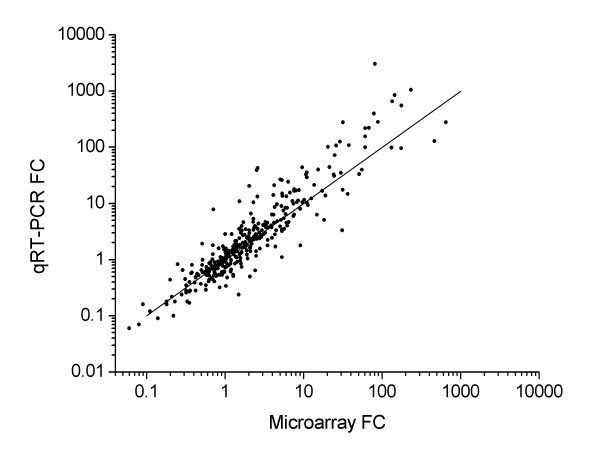
**Postgenomic validation of microarray data.** Correspondence between mean fold change (FC) values obtained by microarray (X) and qRT-PCR (Y) analysis. The diagonal line represents the ideal correspondence trend.

An additional set of three rats per experimental group was subjected to confirmatory analysis of 93 genes by qRT-PCR. This independent validation procedure for microarray analysis results has become standard in genomic studies, although our use of triplicate measurements may have made this step redundant. An excellent correlation was found between microarray and qRT-PCR data (0.89 regression coefficient, n = 1116, Fig. 3). Individual examples can be seen in other Figures (see below). Amplification was not possible with two of the selected genes, namely Defb1 and Htr6. In addition, neither Il6 nor Ifng could be detected in the control samples but both were measured in the colitis groups. Hence, although this is an unmistakable indication of significant induction, a fold change could not be calculated from the qRT-PCR data. The reason for this discrepancy is not known.

Table 1 shows some of the most relevant gene categories affected by TNBS colitis (pre-established gene ontology entries), which differed according to the time point during the inflammatory response. Thus, although the immune response categories predictably dominated at all time points, apoptosis appeared to play a pivotal role at days 2 and 5 while extracellular matrix related genes were especially important at day 7, and a variety of processes and functions were present as leading gene ontology categories at day 14. Manual analysis of the genes showing the most pronounced changes in expression confirmed the importance of immunity/inflammation and tissue remodelling/matrix but also indicated the occurrence of wide changes in transport, metabolism and signalling (see below).

**Table 1:** Gene ontology categories most affected by TNBS colitis at different time points

		Gene ontology category	z value
Day 2	Biological process	Positive regulation of anti-apoptosis	5.033
		Leukocyte chemotaxis	5.199
	Cellular component	Phosphoinositide 3-kinase complex	4.202
	Molecular function	Chemokine activity	5.721
		1-Phosphatidylinositol 3-kinase activity	4.582
		Unfolded protein binding	4.022

Day 5	Biological process	Immune system process	6.507
		Immune response	6.088
		Defence response	6.025
		Response to external stimulus	5.402
		Antigen processing and presentation of peptide antigen *via *MHC class II	5.128
		Lymphocyte mediated immunity	4.962
		Cell death	4.910
		Response to other organism	4.532
		Leukocyte migration	4.506
		Cytokine biosynthetic process	4.484
		Phagocytosis, recognition	4.473
		Positive regulation of lymphocyte differentiation	4.348
		Vasculature development	4.320
		Protein amino acid dephosphorylation	4.289
		Regulation of inflammatory response	4.190
		T cell differentiation	4.091
		Peptide antigen transport	4.044
		Regulation of protein binding	4.001
		Organelle fusion	4.001
	Cellular component	MHC class II protein complex	4.900
		Mitochondrial respiratory chain	4.384
		TAP complex	4.374
	Molecular function	Haematopoietin/interferon-class (D200-domain) cytokine receptor activity	5.107
		Protein tyrosine phosphatase activity	4.871
		Cytokine activity	4.600
		Chemokine activity	4.277
		NADH dehydrogenase activity	4.133
		Peptide antigen-transporting ATPase activity	4.044
		Phosphatidylserine binding	4.001

Day 7	Biological process	Leukocyte chemotaxis	4.936
		Immune system process	4.332
		Response to external stimulus	4.323
		Antigen processing and presentation of exogenous peptide antigen	4.124
	Cellular component	Extracellular matrix part	5.849
		Proteinaceus extracellular matrix	4.931
		Collagen	4.674
		Basement membrane	4.280
		Actin filament	4.115
	Molecular function	Extracellular matrix constituent	4.710

Day 14	Biological process	Defence response	5.420
		Prophyrin catabolic process	5.286
		Immune response	5.082
		Lymphocyte homeostasis	5.029
		DNA damage response, signal transduction by p53 class mediator	4.414
		Glial cell migration	4.414
		Leukocyte activation	4.366
		Inflammatory response	4.362
		RNA destabilization	4.316
		Positive regulation of interferon-gamma production	4.316
		Heme oxidation	4.316
		Vasodilation	4.231
		Cytokine biosynthetic process	4.142
	Cellular component	External side of plasma membrane	5.339
		Costamere	4.316
		B cell receptor complex	4.316
	Molecular function	Double-stranded DNA adenosine deaminase activity	5.286
		Interleukin 1, Type I receptor binding	4.316
		DNA ligase (ATP) activity	4.316
		Heme oxygenase (decyclizing) activity	4.316
		Saccharopine dehydrogenase (NAD+, L-glutamate-forming) activity	4.316
		Thyroid hormone receptor activity	4.316
		Cannabinoid receptor activity	4.316

Non-redundant categories with z values > 4 are shown. A high z value indicates a higher representation of significantly changed genes than expected.

### Time course analysis

The set of genes that was significantly altered by TNBS colitis underwent k-means clustering, a mathematical tool that groups genes with a similar time evolution profile. Most genes clustered to profiles that showed the highest increase (or decrease) at 5 and 7 days, usually peaking at 7 days or staying relatively constant between 2 and 7 days and decreasing or increasing at 14 days (data not shown). However, some atypical profiles were also detected. Thus, the expression of some genes was changed only at day 2 (50 genes, Table 2) or day 14 (57 genes, Table 3) while others were upregulated (Cybb, Nfil3, Cxcr4, Spn, Itgb2) or downregulated (Apob, Amn, Apoa1, Aldob, Cxcl14, Ckm, Slc26a3) at all or most time points. Therefore, somewhat unexpectedly, the time course of gene expression was largely parallel, with few outliers, although there were marked differences in the magnitude of changes. However, as explained above, there was a change over time in the clustering of gene groups according to gene ontology.

**Table 2:** Genes modulated specifically at day 2

Gene symbol	Gene name	FC	SEM
Hla-dmb	Major histocompatibility complex, class II, DM beta	9.13	0.47
Fbxl7_predicted	F-box and leucine-rich repeat protein 7 (predicted)	8.46	3.35
Acot12	Acyl-CoA thioesterase 12	8.19	3.28
Prl8a2	Prolactin family 8, subfamily a, member 2	7.86	2.22
Tmod1	Tropomodulin 1	6.66	0.79
Sds	Serine dehydratase	4.57	0.71
Adcyap1r1	Adenylate cyclase activating polypeptide 1 receptor 1	4.56	0.54
Sp2	Sp2 transcription factor	4.05	0.56
Vmd2l1_predicted	Vitelliform macular dystrophy 2-like protein 1 (predicted)	2.80	0.32
Crh	Corticotropin releasing hormone	2.65	0.26
Fzd4	Frizzled homolog 4 (Drosophila)	2.64	0.14
Igfbp5	Insulin-like growth factor binding protein 5	2.44	0.30
CPG2	CPG2 protein	2.32	0.08
Lss	Lanosterol synthase	2.27	0.08
Reln	Reelin	2.10	0.17
Atcay_predicted	Ataxia, cerebellar, Cayman type (caytaxin) (predicted)	2.05	0.06
Pik3c2g	Phosphatidylinositol 3-kinase, C2 domain containing, gamma polypeptide	2.02	0.25
Fmo2	Flavin containing monooxygenase 2	1.97	0.22
Slc13a5	Solute carrier family 13 (sodium-dependent citrate transporter), member 5	1.90	0.11
Xlkd1_predicted	Extra cellular link domain-containing 1 (predicted)	1.81	0.15
Vps4a	Vacuolar protein sorting 4a (yeast)	1.64	0.03
C1qbp	Complement component 1, q subcomponent binding protein	1.53	0.10
Exosc2_predicted	Exosome component 2 (predicted)	1.52	0.10
Map3k6_predicted	Mitogen-activated protein kinase kinase kinase 6 (predicted)	1.51	0.09
Retnla	Resistin like alpha	0.05	0.03
Csrp2	Cysteine and glycine-rich protein 2	0.10	0.03
Dmrta1_predicted	Doublesex and mab-3 related transcription factor like family A1 (predicted)	0.11	0.00
Rax	Retina and anterior neural fold homeobox	0.13	0.04
Ide	Insulin degrading enzyme	0.14	0.02
Opcml	Opioid-binding protein/cell adhesion molecule-like	0.17	0.01
Wdr22_predicted	WD repeat domain 22 (predicted)	0.18	0.05
Nova1	Neuro-oncological ventral antigen 1	0.19	0.02
Fgf20	Fibroblast growth factor 20	0.21	0.01
Adh6	Alcohol dehydrogenase 6 (class V)	0.22	0.02
Cpg1	Candidate plasticity gene 1	0.22	0.04
Pfkfb2	6-phosphofructo-2-kinase/fructose-2,6-biphosphatase 2	0.27	0.10
Gpha2	Glycoprotein hormone alpha 2	0.31	0.01
Myt1l	Myelin transcription factor 1-like	0.31	0.06
Npffr2	Neuropeptide FF receptor 2	0.33	0.07
Csn2	Casein beta	0.34	0.06
Sybl1	Synaptobrevin-like 1	0.34	0.04
Efcbp2	Neuronal calcium binding 2	0.40	0.06
Cbr3_predicted	Carbonyl reductase 3 (predicted)	0.41	0.03
Alpi	Alkaline phosphatase 1, intestinal, defined by SSR	0.41	0.04
Nrxn1	Neurexin 1	0.43	0.06
Gp2	Glycoprotein 2 (zymogen granule membrane)	0.46	0.03
Cd248_predicted	CD248 antigen, endosialin (predicted)	0.46	0.05
Ppfia3	Protein tyrosine phosphatase, receptor type, f polypeptide (PTPRF), interacting protein (liprin), alpha 3	0.59	0.02
Zfp580_predicted	Zinc finger protein 580 (predicted)	0.62	0.05
Pttg1ip	Pituitary tumour-transforming 1 interacting protein	0.65	0.05

Only annotated genes with a 50% or higher fold change are included. In all cases the FC at days 5–14 was not significantly different from the control (significance at *P *> 0.10 to exclude transcripts not specifically upregulated or downregulated at only day 2).

**Table 3:** Genes modulated specifically at day 14

Gene symbol	Gene name	FC	SEM
Tbx15_predicted	T-box 15 (predicted)	8.00	1.74
Trhr	Thyrotropin releasing hormone receptor	5.67	1.26
Ppp3cc	Protein phosphatase 3 (formerly 2B), catalytic subunit, gamma isoform (calcineurin A gamma)	5.07	3.34
Eif2ak3	Eukaryotic translation initiation factor 2 alpha kinase 3	4.92	2.25
Hrh3	Histamine receptor H3	4.35	1.34
Klf15	Kruppel-like factor 15	3.58	0.39
Gldc_predicted	Glycine dehydrogenase (decarboxylating; glycine decarboxylase, glycine cleavage system protein P) (predicted)	3.40	1.49
Trdn	Triadin	3.18	0.53
Galr1	Galanin receptor 1	3.15	0.04
Mlana_predicted	Melan-A (predicted)	2.30	0.47
Cma1	Chymase 1, mast cell	2.15	0.30
Trerf1_predicted	Transcriptional regulating factor 1 (predicted)	1.91	0.10
Serbp1	Serpine1 mRNA binding protein 1	1.85	0.22
Ltb4dh	Leukotriene B4 12-hydroxydehydrogenase	1.78	0.24
Fgf1	Fibroblast growth factor 1	1.75	0.28
Ace	Angiotensin 1 converting enzyme	1.72	0.33
Eif4a1	Eukaryotic translation initiation factor 4A1	1.68	0.38
Adar	Adenosine deaminase, RNA-specific	1.59	0.12
Ddb1	Damage-specific DNA binding protein 1	1.57	0.05
Mbnl	Muscleblind-like 1 (Drosophila)	1.52	0.10
Lzts1	Leucine zipper, putative tumour suppressor 1	1.52	0.16
Hmga1	High mobility group AT-hook 1	1.51	0.13
Apoa5	Apolipoprotein A-V	0.08	0.01
Strn	Striatin, calmodulin binding protein	0.09	0.05
Olfm3	Olfactomedin 3	0.09	0.01
Cdc2l5	Cell division cycle 2-like 5 (cholinesterase-related cell division controller)	0.11	0.03
Prom2	Prominin 2	0.11	0.02
Snag1_predicted	Sorting nexin associated golgi protein 1 (predicted)	0.11	0.03
Serpina3m	Serine (or cysteine) proteinase inhibitor, clade A, member 3M	0.12	0.02
Ank1_predicted	Ankyrin 1, erythroid (predicted)	0.13	0.00
Hist1h1t	Histone 1, h1t	0.15	0.02
Imp1	Insulin-like growth factor 2, binding protein 1	0.15	0.03
Cacng7	Calcium channel, voltage-dependent, gamma subunit 7	0.16	0.05
Rbm9_predicted	RNA binding motif protein 9 (predicted)	0.19	0.03
Drp2	Dystrophin-related protein 2 A-form splice variant	0.20	0.03
Eif4g1	Eukaryotic translation initiation factor 4 gamma, 1	0.20	0.01
Apob	Apolipoprotein B	0.21	0.07
Wt1	Wilms tumor 1	0.23	0.02
Pqlc2_predicted	PQ loop repeat containing 2 (predicted)	0.25	0.07
Cnr1	Cannabinoid receptor 1 (brain)	0.25	0.02
Lmo3	LIM domain only 3	0.26	0.03
Bai3_predicted	Brain-specific angiogenesis inhibitor 3 (predicted)	0.28	0.04
Sp3	Sp3 transcription factor	0.31	0.02
Apba1	Amyloid beta (A4) precursor protein-binding, family A, member 1	0.32	0.03
Kcnc3	Potassium voltage gated channel, Shaw-related subfamily, member 3	0.44	0.06
Actc1	Actin alpha cardiac 1	0.45	0.06
Wnt11	Wingless-type MMTV integration site family, member 11	0.47	0.09
Il2	Interleukin 2	0.50	0.08
Galnt2_predicted	UDP-N-acetyl-alpha-D-galactosamine:polypeptide N-acetylgalactosaminyltransferase 2 (predicted)	0.57	0.02
Nrg2	Neuregulin 2	0.60	0.04
Cspg4	Chondroitin sulphate proteoglycan 4	0.60	0.02
Enth	Enthoprotin	0.61	0.03
Zfp503_predicted	Zinc finger protein 503 (predicted)	0.66	0.02
Rbm3	RNA binding motif (RNP1, RRM) protein 3	0.66	0.02
Mpp5_predicted	Membrane protein, palmitoylated 5 (MAGUK p55 subfamily member 5) (predicted)	0.66	0.01
Prg1	Plasticity related gene 1	0.67	0.02
Csnk1d	Casein kinase 1, delta	0.67	0.06

Only annotated genes with a 50% or higher fold change are included. In all cases the FC at days 2 – 7 was not significantly different from the control (significance at *P *> 0.10 to exclude transcripts that were not specifically upregulated or downregulated at only day 14).

### Genes involved in the inflammatory response

**Figure 4:**
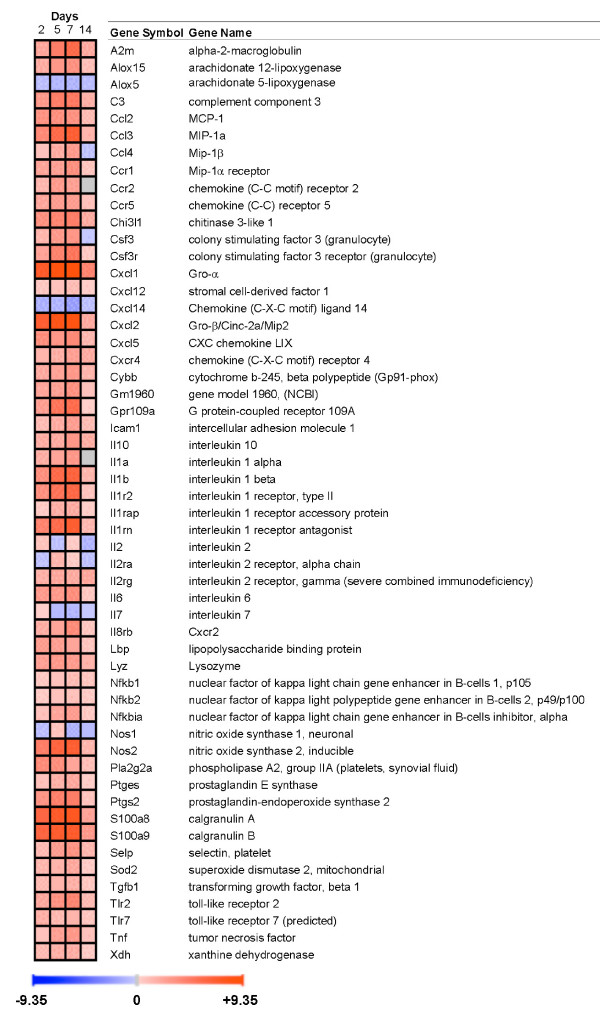
**Behaviour over time of genes involved in inflammation and immune response. **Upregulated genes are shown in red and downregulated genes in blue. Gene expression was considered significantly changed by inflammation at p < 0.05 after analysis of variance followed by Tukey post-hoc tests and Benjamini & Hochberg false discovery rate correction.

**Figure 5:**
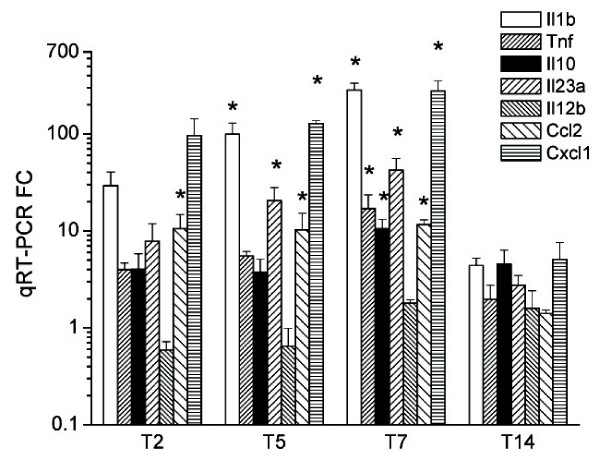
**Postgenomic validation of selected inflammation genes by qRT-PCR.** *P < 0.05 vs. control.

Many of the most severely affected genes were those directly involved in the inflammatory response, as expected (Fig. 4, see also Fig. 5 for validated genes). Chemokines were especially prominent, including neutrophil chemokines and other leukocyte-attractant molecules. Interleukin 1 and related TLR2 pathways were highly affected, and both converged in the activation of NF-κB. Nfkbia (encoding IκB-α) was markedly increased, indicating a trend to limit activation of this pathway. The intestinal inflammatory marker Alpl [18] was prominently upregulated, unlike Alpi and Alpi2, the intestinal isoforms of alkaline phosphatase (data not shown).

The prostaglandin biosynthetic pathway was strongly activated by TNBS through the concerted induction of Pla2g2a, Ptgs2, and Ptges, especially during the chronic phase (confirmed by qRT-PCR, data not shown). Other affected genes are related to cell adhesion, bacteria binding or reactive oxygen species generation. There were also marked changes in the expression of numerous transcripts involved in antioxidative defence, most of which were downregulated.

In addition, close examination of the expression level of a number of genes that act as inflammatory cell markers indicated the nature of the inflammatory infiltrate. Thus, TNBS colitis was characterized by an absence of significant changes in markers of T cells (Thy1, Tcrb, Tcrg, Zap70, Lck), B cells (Ptprc -B220-, Ms4a1 -Cd20-, Cd22, Cd79b) and NK cells (Baat, Ncam1 -Cd56-, B3gat1 -Cd57-). In contrast, several neutrophil/macrophage markers such as S100a8, Itgb2 (Cd18), Cd68_predicted, Csf1r, Cybb, Csf2rb1 and Lcn2 were significantly increased by inflammation, especially at days 5 and 7.

### Genes involved in metabolism

**Figure 6:**
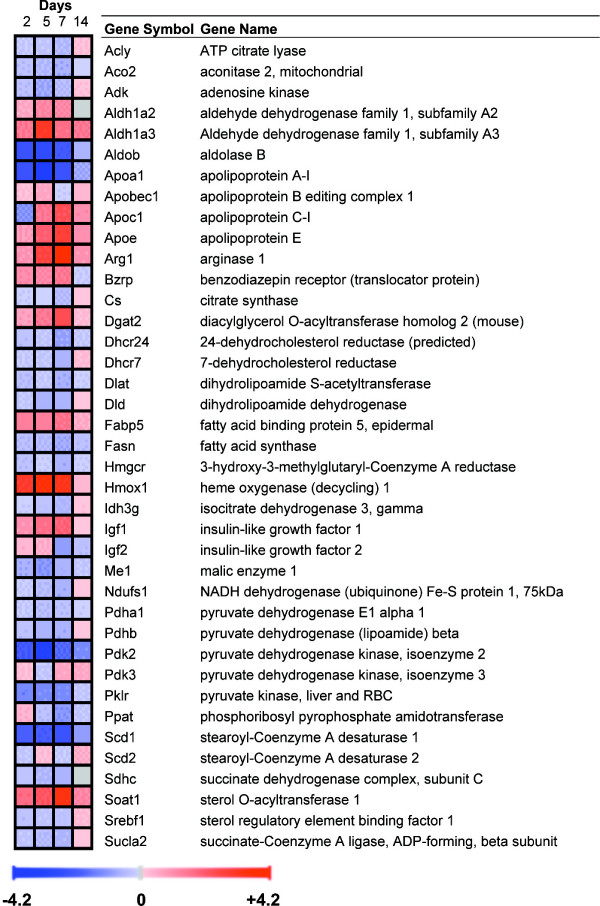
**Behaviour over time of genes involved in metabolism.** Upregulated genes are shown in red and downregulated genes in blue. Gene expression was considered significantly changed by inflammation at p < 0.05 after analysis of variance followed by Tukey post-hoc tests and Benjamini & Hochberg false discovery rate correction.

**Figure 7:**
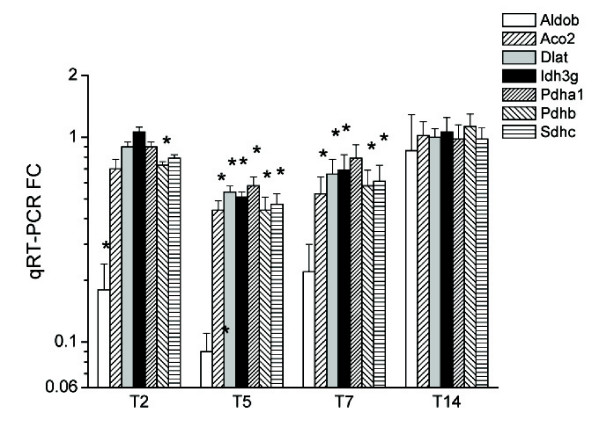
**Postgenomic validation of selected metabolic genes by qRT-PCR.** *P < 0.05 vs. control.

Metabolism-related genes were highly affected (mostly decreased) by TNBS-induced colitis (Fig. 6 and also Fig. 7 for validated genes). They included genes participating in glycolysis, like Aldob and many different subunits of the pyruvate dehydrogenase complex (Dlat, Dld, Pdha1, Pdhb). Several enzymes of the Krebs cycle were also changed, e.g., Idh3g, Aco2, Sucla2 and Cs. The respiratory chain genes Ndufs1 and Sdhc and several genes encoding cytochrome isoforms were also downregulated.

With regard to lipid metabolism, the fatty acid biosynthetic enzymes Fasn and Scd1 were inhibited, as was the related gene Acly. Several transcripts related to cholesterol biosynthesis and transport were also affected (Hmgcr, Srebf1, Bzrp, Dhcr7, Dhcr24, Apoa1, Abca1). In addition, both ApoE and the apolipoprotein B mRNA editing gene Apobec1 were upregulated, suggesting a higher lipoprotein biosynthesis, although ApoE may also play a protective role against lipopolysaccharide [19]. A number of other metabolic pathways appeared to be altered by TNBS colitis, including purine *de novo *biosynthesis, arginine and heme catabolism.

### Genes involved in signalling

**Figure 8:**
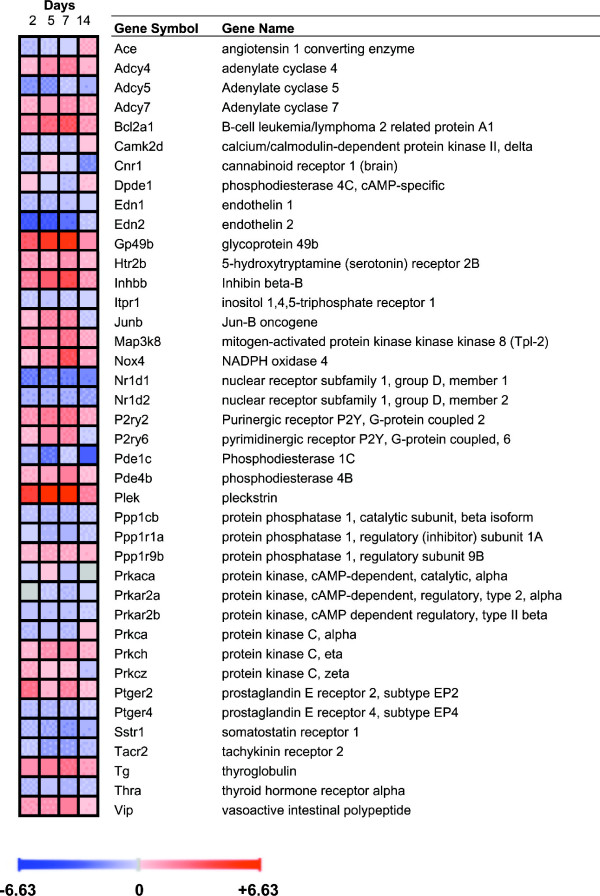
**Behaviour over time of genes involved in cell signalling.** Upregulated genes are shown in red and downregulated genes in blue. Gene expression was considered significantly changed by inflammation at p < 0.05 after analysis of variance followed by Tukey post-hoc tests and Benjamini & Hochberg false discovery rate correction.

Many transcripts involved in cell signalling were changed by TNBS colitis, including genes encoding proteins that participate in cAMP/protein kinase A and calcium/protein kinase C pathways, phosphatases, various regulatory proteins and a number of transmitter/hormone receptors, including P2ry6, Htr2b, Prlr, Sstr1, Tacr2 and Thra. In addition, Ace was modestly increased only on day 14, suggesting a possible local increase in angiotensin II production in the healing colonic tissue (Fig. 8).

The signalling-related genes that exhibited the highest changes in expression included Gp49b and Plek. Gp49b encodes an immunoglobulin-like receptor expressed in myeloid cells that appears to counter-regulate the cytokine and chemokine attraction of neutrophils [20]. The function of Plek appears to be related to the regulation of macrophage phagocytosis [21].

### Genes involved in transport

**Figure 9:**
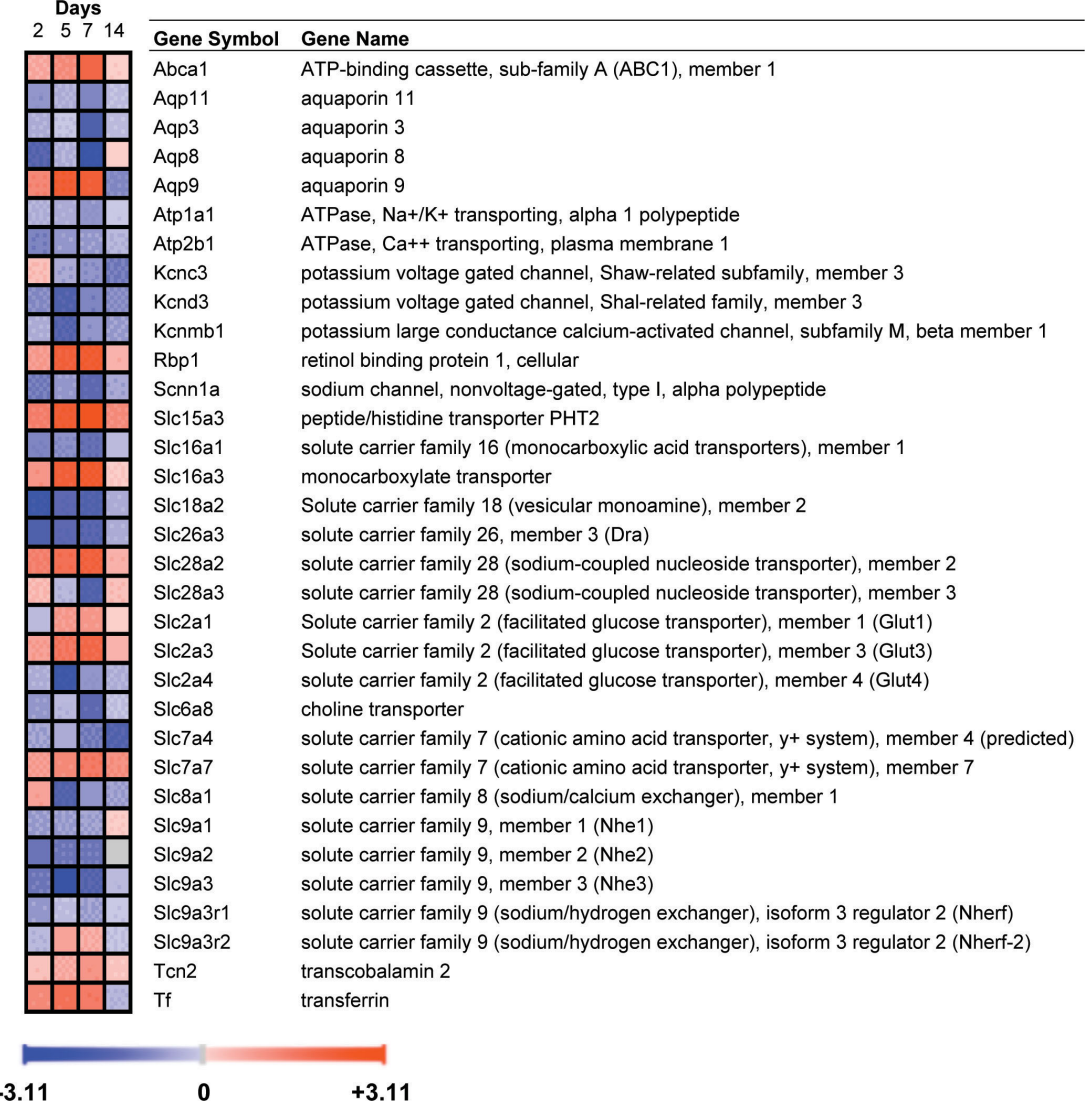
**Behaviour over time of genes involved in transport.** Upregulated genes are shown in red and downregulated genes in blue. Gene expression was considered significantly changed by inflammation at p < 0.05 after analysis of variance followed by Tukey post-hoc tests and Benjamini & Hochberg false discovery rate correction.

**Figure 10:**
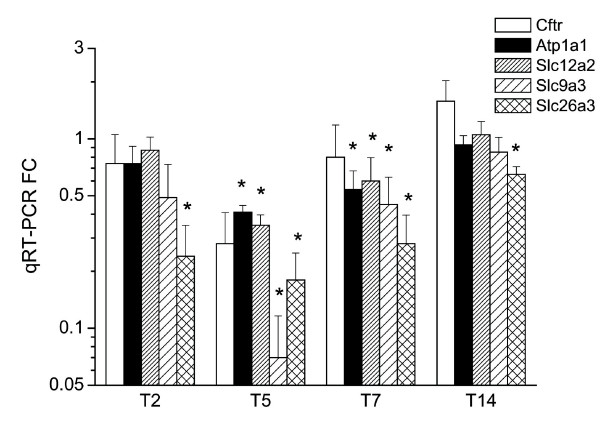
**Postgenomic validation of selected ion transport genes by qRT-PCR.** *P < 0.05 vs. control.

TNBS colitis influenced the expression of many transport-related transcripts, including genes that participate in ionic transport, e.g. Slc9a2, Slc9a3 (Nhe2-3), Scnn1a and Slc26a3 (Dra), which mediate NaCl absorption, and the Na^+^/K^+ ^pump subunit Atp1a1 and Slc12a2 (Nkcc2), all of which were inhibited. However, the chloride channel Cftr was not affected. Three aquaporins (Aqp3, 8 and 11) were also repressed in TNBS colitis, whereas Aqp9 was upregulated. Many other nonionic transporters and ionic channels were altered (Fig. 9 and Fig. 10 for validated genes). Interestingly, there was upregulation of Slc7a7, which is involved in the basolateral transport of arginine, lysine and ornithine by epithelial cells and activated monocytes [22], suggesting increased availability of iNOS substrate.

### Genes involved in tissue remodelling

**Figure 11:**
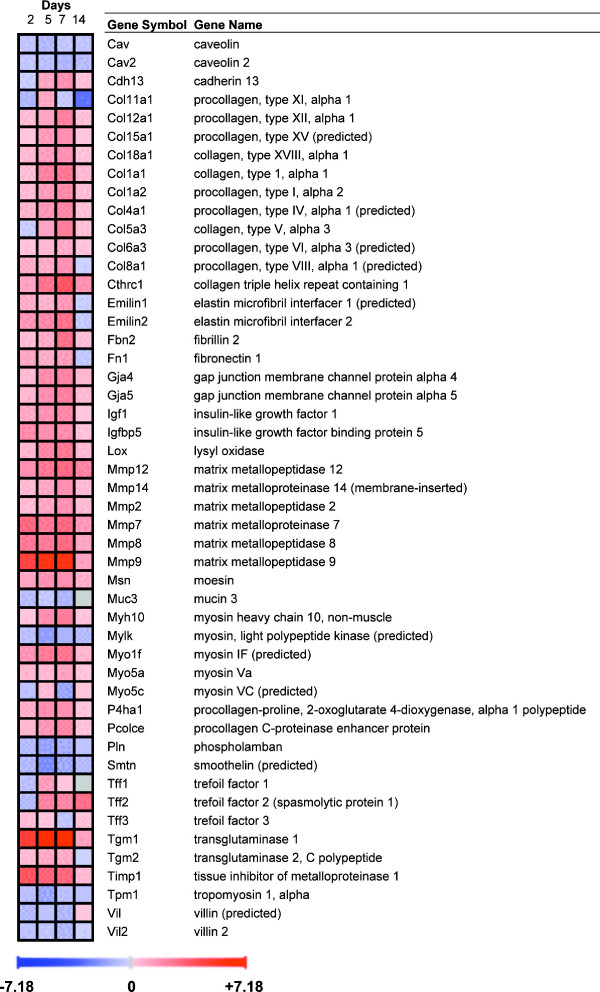
**Behaviour over time of genes involved in tissue remodelling.** Upregulated genes are shown in red and downregulated genes in blue. Gene expression was considered significantly changed by inflammation at p < 0.05 after analysis of variance followed by Tukey post-hoc tests and Benjamini & Hochberg false discovery rate correction.

**Figure 12:**
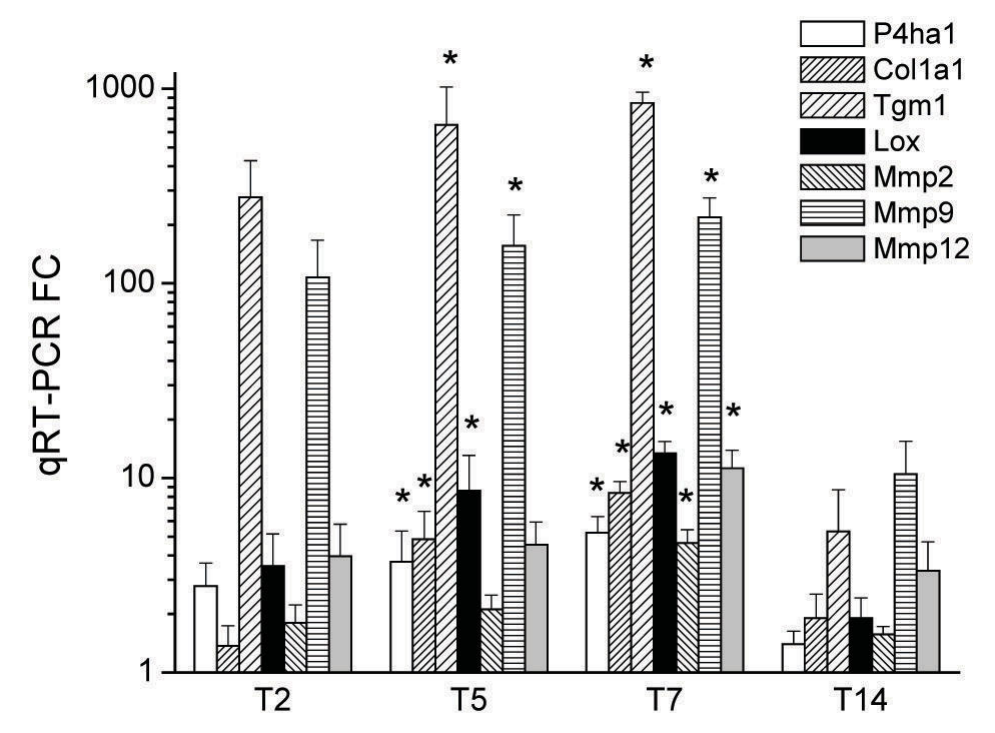
**Postgenomic validation of selected tissue remodeling genes by qRT-PCR.** *P < 0.05 vs. control.

Following the gene ontology analysis, a manual search of the most severely affected genes confirmed the dramatic changes in the expression of many genes involved in matrix deposition, muscle plasticity and angiogenesis (see Figs. 11 and 12 for validated genes). These include Igf1 (validated) and Igfbp5, genes that may regulate tissue remodelling by increasing collagen synthesis and cell proliferation. Several procollagen/collagen isoforms were increased, as well as genes involved in collagen processing and synthesis or in collagen and elastin fibre cross-linking (Lox, Tgm1 – both validated-). A number of metalloproteases and multiple cytoskeletal genes were increased, especially during the chronic phase.

## Discussion

The main features of the TNBS model of colitis have been well defined. Ethanol causes direct toxic damage to the epithelium, which in turn grants TNBS access to the colonic mucosa, where it acts as a hapten. Although the toxic effect of ethanol is a requisite for TNBS to act effectively, the immunogenic nature of the 'chronic' phase has been unequivocally established by various authors [23-25]. Without TNBS, ethanol produces only a short-lived inflammatory reaction that resolves without sequelae and was therefore not of interest in the present study. During the 'chronic' phase, TNBS rat colitis shares a number of features in common with IBD, e.g., transmural inflammation (Crohn's disease only), abnormal ion transport, diarrhoea, fibrosis and abnormal intestinal motility. Furthermore, TNBS rat colitis is amenable to treatment with corticoids, sulfasalazine or tacrolimus, making it an attractive model for the preclinical testing of putative IBD drugs. Our aim was to perform a detailed genomic characterization of the model.

The TNBS colonic inflammatory response was characterized by a marked increase in the expression of multiple genes involved in inflammation/immunity, including cytokines, chemokines, adhesion molecules, eicosanoid-related genes, and a number of effectors and cell markers. There was a dramatic increase in Il1b, Il1a, Il6, Tgfb1 and Tnf and in various chemokine and chemokine receptors. In addition, a nonsignificant increase was observed in the pivotal Il23a and Ifng cytokine genes, which was found to be significant after re-examination using the more sensitive qRT-PCR. Although no protein data are avaliable, it is tempting to speculate that the combined upregulation of Il23a, Il6 and Tgfb1 are indicative of Th17 induction [26]. In fact, although Il17a was not represented in the microarray, qRT-PCR findings showed an increase in the mRNA levels (data not shown), suggesting the involvement of Th17 cells in rat TNBS colitis. A contribution of Th1 cells is also likely in view of the Ifng upregulation and lack of Il4 changes, although Il12a and Il12b were not augmented. Our group [27] and other authors [28,29] previously reported an increase in the IFN-γ or IFN-γ/IL-4 protein ratio in this model. A staggering increase was observed in some of the genes, e.g. S100a9/S100a8, suggesting that they may be highly sensitive markers to follow intestinal inflammation, as previously observed in humans [30,31]. These genes encode proteins expressed by macrophages and neutrophils and are involved in bacterial defence, chemotaxis and signalling [32,33].

Analysis of the microarray data offered insights into the nature of the inflammatory infiltrate. TNBS colitis was previously characterized at this level [25]. In brief, macrophages and neutrophils are initially recruited to the colonic mucosa and submucosa, followed by a more predominant role of lymphocytes in later stages of colitis, which are characterized by progressive healing, reepithelialization, crypt enlargement and prominent fibrosis [25]. The predominance of neutrophils/macrophages in the inflammatory response was confirmed in our study by the global increase in gene markers of these cell types and the absence of effects on B, T and NK cells (at significance level of p < 0.05). It should be noted that the sample used for genomic analysis did not include lymphoid follicles, in which lymphocytes accumulate in this model [25], which explains the lack of a significant increase. However, gene ontology analysis indicated that lymphocyte differentiation was already prominent at day 5.

Our data also revealed important changes that were not directly linked to inflammation/immunity. Thus, marked alterations were found in metabolism-related genes, indicating a reduced functionality of numerous biochemical pathways, including glycolysis, purine biosynthesis, Krebs cycle, cholesterol biosynthesis and transport, fatty acid biosynthesis and respiratory chain. Taken together, these data demonstrate a decrease in biosynthesis of macromolecules, an increase in catabolism, and a decrease in aerobic and anaerobic respiration. Therefore, mucosal cells may be have inadequate energy resources during the inflammatory response. Similar changes have been reported in IBD patients [34] and in experimental colitis [35].

A number of genes encoding ion transporters were affected by TNBS colitis, including: Atp1a1, encoding the catalytic subunit of the Na^+^/K^+ ^pump; Scnn1a, encoding the alpha polypeptide of the epithelial sodium channel; Nhe1, encoding the housekeeping Na^+^/H^+ ^exchanger; and Slc12a2, encoding Nkcc2, the Na^+^/K^+^/Cl^- ^cotransporter. These were generally downregulated, as were the transporters involved in NaCl absorption, e.g., Nhe2, Nhe3, and Slc26a3. However, Cftr was unchanged. These modifications are consistent with severe alterations of ionic transport, as demonstrated in IBD and animal models [17,36,37].

A striking finding of our analysis was the vast number of upregulated genes involved in tissue remodelling in TNBS colitis, generally during the chronic phase, as indicated by the gene ontology analysis. The chronic phase is characterized by mucosal wound healing and submucosal fibrosis and by the scarring and deposition of excess muscular tissue, extending through day 14 and eventually leaving sequelae [5]. These features are often detectable by the naked eye as colonic shortening, deformation and rigidity. Two features are related to the intestinal fibrosis suffered by IBD patients: an increase in collagen synthesis by smooth muscle cells, fibroblasts and myofibroblasts, and an increase in muscle layer thickness. IGFBP-5 expression is known to increase in ulcerative colitis, while IGF-1 and TGF-β_1 _expression is known to increase in both ulcerative colitis and Crohn's disease [38,39]. These molecules have been related to extracellular matrix remodelling and may therefore be relevant to the fibrosis observed in IBD. Our results are in agreement with these IBD findings, showing an increase in Igf1, Igfbp5 and Tgfb1 expression in the inflamed colon. Hence, the TNBS model may be appropriate for the study of molecular mechanisms implicated in the development of fibrosis in intestinal inflammation. In addition, expressions of procollagen/collagen genes, most notably Tgm1, and of several enzymes related to collagen processing were increased in the TNBS colitic animals.

Our results include some intriguing findings, including: the severe repression of nuclear receptors Nr1d1 and Nr1d2, involved in circadian expression patterns and recently shown to be regulated by heme [40]; the synthesis and marked upregulation of Tg in the inflamed colon; and the downregulation of the thyroid receptor. Further experiments are warranted to clarify the role of these changes in colonic inflammation.

We were especially interested in establishing a correlation between the known characteristics of the model and the time course of gene expression. Because the time points selected cover all stages of the TNBS-induced inflammatory response, from acute (2 d) to chronic (5–7 d) and healing/recovery (14 d) phases, we expected to find substantial differences in the pattern of expression. For instance, genes involved in inflammation/immunity and tissue regeneration were expected to predominate in the early and late stages, respectively. However, the vast majority of transcripts followed a common trend, namely a change of expression that was maximal at days 5 and 7, when colitis is most prominent, and was normal (or almost normal) at day 14. Hence, there is a close correlation between the pathological features of TNBS colitis and the changes observed in the transcriptome. The genes that were up- or downregulated only at days 2 or 14 did not conform to any specific category. Some of the transcripts involved are of interest. Thus, Retnla, which encodes resistin-like alpha, which is dramatically repressed soon after colitis induction, may be involved in monocyte activation, as is the beta isoform [41]. Both Ace and Cma1, which encode enzymes with angiotensin I cleaving capacity, are induced specifically at day 14. Some specific receptors involved in cellular signalling are differentially regulated, such as Galr1, Hrh3, Npffr2 and Cnr1. Further analysis is warranted to explore the implications of these findings.

Despite using a technical approach (triplicate samples per time point) that meets or surpasses common standards in genomic studies, we carried out more than 1100 qRT-PCR determinations in 15 additional samples for *a posteriori *data validation. As expected, the overall correlation was excellent. In some cases, microarray analysis appeared to be less prone to pick up statistically significant variations, i.e. it was less sensitive than qRT-PCR. The fact that we examined four different time points adds to the complexity of the analysis, since a marked change at one time point may not be enough in many cases to reach significance in the ANOVA. Further validation is provided by the fact that the changes (or lack thereof) observed in many genes, including Il1b, Il1rn, Cftr, Tnf, Alpl, Alpi, Ifng, Il4, Il6, Pgts2, Nos2 and Ccl2, among many others, are in line with previous observations from our own group, either at the RNA or protein level [12,18,27].

Previous studies have examined changes in the transcriptome in intestinal inflammation, especially in mouse colitis elicited by TNBS and other manoeuvres [6-10]. These studies are somewhat limited because they either use small microarrays, examine animals shortly after colitis induction (consistent with acute but not chronic colitis), offer limited or no postgenomic validation or use longitudinal tissue samples. One of these studies addressed the time course of gene expression, but it studied recovery from repeated TNBS challenges [9]. In general, the changes in gene expression documented in the present study are much stronger and wider than those found in previous reports. For example, 175 genes were differentially expressed in the above study in compared to the thousands of genes in our investigation. On the other hand, there have been several human IBD microarray studies [30,31,42-47], which have detected some key gene expression changes associated with human intestinal inflammation, including some specifically linked to either ulcerative colitis or Crohn's disease [47]. However, it is poorly understood how genomic data are related to IBD pathology and therapy, partly because of the variability inherent to human studies and partly because of methodological differences [48]. For instance, the most important source of variation in gene expression measurement (54%) is the microarray platform itself [49], and all of the above studies used different arrays. There are also differences in design and especially in sample selection, i.e., individual vs. pooled or mucosal vs. full thickness. Nevertheless, there are some similarities with the rat TNBS model, including increases in Chi3l1, Mmp3, 10 and 12, Apoe, S100a8, Ltb, Sod2, Cxcl1, Cxcl2, Il6 and many other genes.

## Conclusion

We have characterized the rat TNBS model of IBD at the genomic level, obtaining novel data on individual genes, metabolic pathways and biological functions that are altered during colitis. These findings establish a basis for further mechanistic studies on drug action and research into the pathophysiology of intestinal inflammation. The full genomic database will serve as a reference standard for all future investigations using this model and for comparisons with other animal models of colitis, an approach that is currently being applied in our laboratory.

## Abbreviations

IBD: inflammatory bowel disease; IL: interleukin; qRT-PCR: quantitative real time PCR; TNBS: trinitrobenzenesulphonic acid.

## Authors' contributions

FSMLH and OMA conducted the animal experiments, coordinated the bioinformatic analysis of the microarray and qRT-PCR data and drafted the manuscript. FSMLH, OMA, MDS and AZ conceived the study. All authors contributed to the interpretation of the data and have read and approved the final manuscript.
